# Insight Into the Multiple Branches Traits of a Mutant in *Larix olgensis* by Morphological, Cytological, and Transcriptional Analyses

**DOI:** 10.3389/fpls.2021.787661

**Published:** 2021-12-21

**Authors:** Kewei Cai, Xueyan Zhou, Xiang Li, Ye Kang, Xiaoming Yang, Yonghong Cui, Guangyan Li, Xiaona Pei, Xiyang Zhao

**Affiliations:** ^1^College of Forestry and Grassland, Jilin Agricultural University, Changchun, China; ^2^State Key Laboratory of Tree Genetics and Breeding, Northeast Forestry University, Harbin, China; ^3^Seed Orchard of Siping, Siping, China

**Keywords:** mutant plants, multiple branches, cytological structure, RNA-seq, plant hormones, *Larix olgensis*

## Abstract

*Larix olgensis* is a tall deciduous tree species that has many applications in the wood fiber industry. Bud mutations are somatic mutations in plants and are considered an ideal material to identify and describe the molecular mechanism of plant mutation. However, the molecular regulatory mechanisms of bud mutations in *L. olgensis* remain unknown. In this study, dwarfed (or stunted), short-leaved, and multi-branched mutants of *L. olgensis* were found and utilized to identify crucial genes and regulatory networks controlling the multiple branch structure of *L. olgensis*. The physiological data showed that the branch number, bud number, fresh and dry weight, tracheid length, tracheid length-width ratio, inner tracheid diameter, and epidermal cell area of mutant plants were higher than that of wild-type plants. Hormone concentration measurements found that auxin, gibberellin, and abscisic acid in the mutant leaves were higher than that in wild-type plants. Moreover, the transcriptome sequencing of all samples using the Illumina Hiseq sequencing platform. Transcriptome analysis identified, respectively, 632, 157, and 199 differentially expressed genes (DEGs) in buds, leaves, and stems between mutant plants and wild type. DEGs were found to be involved in cell division and differentiation, shoot apical meristem activity, plant hormone biosynthesis, and sugar metabolism. Furthermore, bZIP, WRKY, and AP2/ERF family transcription factors play a role in bud formation. This study provides new insights into the molecular mechanisms of *L. olgensis* bud and branch formation and establishes a fundamental understanding of the breeding of new varieties in *L. olgensis*.

## Introduction

Somatic mutations are mutations that occur in an organism’s cell other than in a gamete, germ cell, or gametocyte (Jung et al., 2015). Most somatic mutations have no phenotypic effect, but some somatic mutations in plants can cause certain changes in leaf and branch shapes (Tian et al., 2021a). Somatic mutations have proved to be an important part of functional genetics research in model plants (Chatelet et al., 2007). Apart from the artificial mutations induced in model plants, naturally occurring mutations are widely found in most species (Koornneef et al., 2004; Liu et al., 2009). Moreover, somatic mutations, which are not usually transmitted to the offspring, are different from germline mutations, which can be passed on to the descendants of an organism (Liu et al., 2021). This distinction may be blurred in plants, which lack a dedicated germline, and can propagate asexually through grafting, cutting, and other mechanisms (Foster and Aranzana, 2018).

Bud mutation is a type of somatic mutation in plants, which refers to changes in the genetic material in meristem cells of the bud during cell division (Leng et al., 2021). The plants with bud mutation show corresponding changes in external morphology, internal structure, physiology, and biochemistry and show different characteristics from the original plant, such as lateral shoot, inflorescence or flower, and fruit (Liu et al., 2009; Foster and Aranzana, 2018). Among them, branching is a significant phenotypic change in bud mutation plants (Zhao et al., 2021). Branching is an extremely complex biological process controlled by a variety of factors, such as genetics (Brackmann and Greb, 2014), hormones (Domagalska and Leyser, 2011; Xu et al., 2015), and the environment (Djennane et al., 2014). In addition, the special traits generated due to bud mutation can be stably maintained by asexual reproduction methods such as grafting and cutting and can be inherited by the offspring (Chen et al., 2019). Bud mutation preserves the desirable parent plant qualities and provides valuable new characteristics (Foster and Aranzana, 2018). Therefore, as a source of plant variation, bud mutation not only selects new varieties directly, but also provides new germplasm for hybrid breeding, and thereby making it a simple and effective method for breeding new varieties (Wang et al., 2017; Du et al., 2020).

*Larix olgensis* belongs to the family Pinaceae, which is a fast-growing tree with strong adaptability, high ornamental value and excellent wood properties. Its wood is widely used for construction purposes, shipbuilding, railway track building, and paper making (Zhang et al., 2021). In our study, we have identified bud mutants in *L. olgensis* which showed stunted growth, shorter leaves, and multiple branches in comparison with wild-type plants. These mutants show potential characteristics to generate new varieties of this ornamental plant. However, the molecular regulatory mechanisms of bud and branching formation in *L. olgensis* remain unknown, and therefore, these mutants provide an excellent resource to investigate the physiological and molecular mechanisms in this plant.

In this study, wild-type and mutant plants (named N′ and V′, respectively) from *L. olgensis* parent trees were collected, and N′ and V′ branches were grafted onto the same type of rootstocks, and they were cultured under the same conditions. After 1 year, N′ and V′ grafted seedlings (named N and V, respectively) with the same growth vigor were selected preserved. The mechanism of bud formation was investigated using N′, V′, N, and V as plant materials. The growth and physiological indicators of N, V, N′, and V′ were measured, including the branch number, bud number, fresh and dry weight, tracheid length and width, epidermal cell area, and plant hormone content. Additionally, to investigate the molecular mechanism of bud mutations, we performed transcriptome sequencing of wild-type and mutant plants and identified crucial genes and regulatory networks controlling the multiple branch structure of *L. olgensis*. The results provide new insights to understand the molecular mechanisms of *L. olgensis* mutant plants and provide basic information and technical support for the breeding of new varieties of *L. olgensis*.

## Materials and Methods

### Plant Materials

The plants were cultivated in the forest seed orchard of Siping City (124°10΄E; 43°05΄N), Jilin Province, China. In July 2019, wild-type and mutant plants branches from *L. olgensis* parent trees were collected and grafted onto the same type of rootstocks, and they were cultured under the same conditions. In July 2020, wild-type and mutant plant branches from *L. olgensis* parent tree (named N′ and V′, respectively) were randomly collected, and wild-type and mutant plant grafted seedlings (named N and V, respectively) with the same growth vigor were selected preserved. Each sample contained five biological repeats to measure the physiological and biochemical indexes. Buds, leaves, and stems of wild-type and mutant plant branches from *L. olgensis* parent tree were collected, with each sample containing three biological repeats. These samples were quickly transferred into packing tubes and stored at −80°C for transcriptomic sequencing analysis.

### Measurement of Growth Traits

The primary and secondary branches of N, V, N′, and V′ annual branches were distinguished, and the number of branches was counted. Branches with lengths greater than 10 cm from N, V, N′, and V′ annual branches were selected, and the number of bud points within 10 cm of each branch was counted. The length of the primary and secondary branches of N, V, N′, and V′ annual branches were measured using a ruler with 0.1 cm precision. The width of the primary and secondary branches in N, V, N′, and V′ annual branches was measured using a vernier caliper with 0.01 mm precision (Each sample contained 5 biological repeats). The needles were randomly selected from N′, V′, N, and V. The top and bottom length and width of needles were measured using a vernier caliper with 0.01 mm precision, and the average value was calculated as the needle width. The needles length-width ratio was also calculated (Each sample contained 30 biological repeats). The needles and branches were randomly selected from N′, V′, N, and V. The fresh weight was recorded using an electronic balance, and the sample was dried at 105°C using an electric blast drying oven for 48 h, and the dry weight was recorded thereafter (each sample contained 5 biological repeats). SPSS 26.0 software was used for the statistical analysis of all data, and significant differences between the samples were determined using Student’s t test. Differences were considered statistically significant at *p* < 0.05.

### Cytological Observation and Analysis

Stem segments from N′, V′, N, and V were treated using the Jeffrey segregation process (Mu et al., 2009). The tracheid length and width were measured using a stereomicroscope and the tracheid length-width ratio was calculated. The buds, leaves, and stems from N′ and V′ were studied using an improved paraffin section technique (Ruzin, 1999; Guo et al., 2019). The samples were dehydrated in ethanol solutions of different concentrations and then treated with dimethyl benzene and embedded in paraffin. The samples were sectioned at 10 μm thickness using a slicer and stained with safranine. The glass slides were observed using an optical microscope, and the typical structures were selected for photographing.

The sampled stems for N′ and V′ were fixed using formalin-acetic acid-alcohol (FAA) fixative for more than 24 h, washed twice with phosphate-buffered saline buffer and distilled water, dehydrated with different ethanol concentrations, and again dehydrated with absolute ethanol twice. The materials were critical-point dried and sprayed with gold. The treated materials were observed and photographed using the NeoScope JCM-5000 scanning electron microscope. Moreover, tracheid double wall thickness, tracheid inner diameter, pith cells’ inner diameter, and pith cells’ double wall thickness were measured under different multiples of optical and scanning electron microscopes.

### Measurement of Physiological Traits

Photosynthesis rate (Pn, μmol m^−2^ s^−1^), transpiration rate (Tr, mol m^−2^ s^−1^), intercellular carbon dioxide concentration (Ci, μmol mol^−1^), and N and V stomatal conductance (Gs, mol m^−2^ s^−1^) were measured using LI-6400 Portable Photosynthesis System in sunny days. The instantaneous water use efficiency (Wue, μmol mol^−1^) was calculated using the following formula: water use efficiency (WUE) = Pn/Tr. The determination time was 9:00–11:00 AM; light intensity, 1,000 μmol m^−2^ s−1; carbon dioxide concentration, 380 μmol mol^−1^; and other environmental factors were without special controls.

The concentrations of abscisic acid (ABA), auxin (IAA), ethylene, and gibberellin (GA) in needles and shoots of N, V, N′, and V′ were measured using ELISA and high-performance liquid chromatography, respectively, in July and September by the Shanghai Enzymatic Biotechnology Company Ltd.

### RNA Extraction, Cdna Synthesis and Sequencing

Plant total RNA isolation kit (TaKaRa, Beijing, China) was used to extract total RNA from samples, following the manufacturer’s instructions, and different samples were subjected to three biological repeats. After qualification using a bioanalyzer (2,100, Agilent, United States), 20 μg of each sample was used for cDNA library construction. cDNA synthesis was based on a previous study (Li et al., 2021). All of the samples were sequenced using the Illumina HiSeq sequencing platform (Hiseq^™^ 2000, Illumina, United States). The raw reads were filtered using the Fastp software (version 0.12) to obtain high-quality clean reads (Chen et al., 2018). The reference sequence was assembled using the Trinity software (version 2.0.6; Grabherr et al., 2011). The longest transcript at each locus was considered a unigene, and the unigene IDs were automatically generated by the software (Yang et al., 2019).

### Sequence Annotation and Differential Expression Gene Analysis

To obtain comprehensive gene function information, the assembled unigene sequences were aligned with sequences from public databases, including COG (The database of Clusters of Orthologous Groups of proteins; Tatusov et al., 2000), KOG (euKaryotic Orthologous Groups; Koonin et al., 2004), KEGG (Kyoto Encyclopedia of Genes and Genomes; Kanehisa et al., 2004), GO (Gene Ontology; Ashburner et al., 2000), Pfam (Protein family), Swiss-Prot (A manually annotated and reviewed protein sequence database; Apweiler et al., 2004) and Nr (non-redundant protein sequence database; Deng et al., 2006), using BLAST (Altschul et al., 1997) and corresponding annotations were obtained. All clean reads were mapped to reference sequences using RSEM software (version 1.2.26; Li and Dewey, 2011), and fragments per kilobase of transcript per million fragments mapped reads were used to calculate the expression level of each gene (Trapnell et al., 2010). Differentially expressed genes (DEGs) were identified between the groups using the DESeq2 R package (version 3.11; Love et al., 2014). The value of *p* was adjusted using the Benjamini and Hochberg false discovery rate (Benjamini and Hochberg, 1995). The genes with *q*-value < 0.05 and |Fold Change (FC)| ≥ 1 were considered significantly differentially expressed. GO functional annotations of DEGs were searched using EggNOG 5.0,^1^ and the results were plotted using the annotation numbers in the WEGO 2.0 analysis.^2^ KEGG pathway annotations were performed using EggNOG 5.0, and the results were plotted using the OmicShare tools.^3^

### Quantitative Real-Time Polymerase Chain Reaction Validation

The plant total RNA isolation kit (TaKaRa, Beijing, China) was used to extract total RNA from samples. cDNA was synthesized from RNA sample using a cDNA Synthesis Kit (Takara, Kyoto, Japan), according to the manufacturer’s instructions. Quantitative real-time polymerase chain reaction (qRT-PCR) was performed using ABI 7500 RT PCR system. The Primer Premier 5.0 was used to design primers, and IDH was used as a reference gene. The PCR reaction protocol was as follows: 94°C for 30 s, 45 cycles of 94°C for 5 s, 60°C for 35 s, 95°C for 15 s, 60°C for 1 min, followed by 95°C for 15 s. The relative expression level was calculated according to the 2^−ΔΔCT^ method. In addition, each sample contained three biological repeats.

## Results

### Changes in Growth and Biomass of Mutant Plants

**Figure 1:**
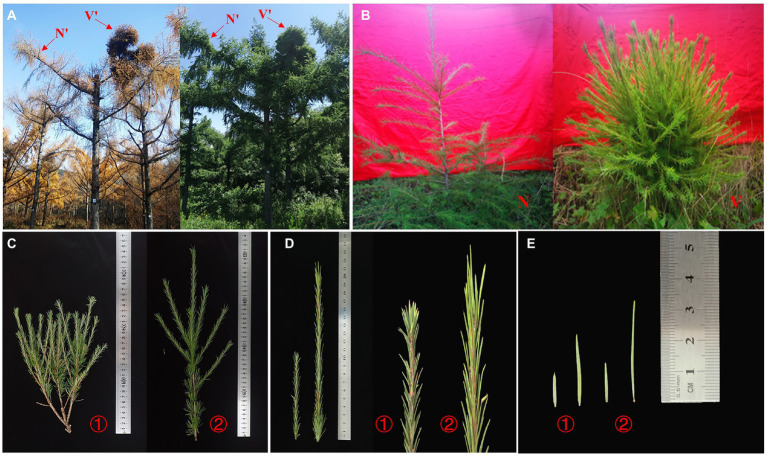
Phenotypes of wild-type and mutant plants. **(A)** Phenotypic structure of wild-type branches (N') and mutant plant branches (V') from *Larix olgensis* parent trees. **(B)** Phenotypic structure of grafted seedlings of wild-type and mutant plants (N and V). **(C)** Perennial branches of wild-type and mutant plants of *L. olgensis* parent tree. **(D)** Annual branches of wild-type and mutant plants of *L. olgensis* parent tree. **(E)** Needles of wild-type and mutant plant of *L. olgensis* parent tree. ① wild-type plant; ② mutant plant.

In this study, V and V′ were selected for stunted growth, shorter leaves and multiple branches, and then, the phenotypes differences of plant materials N, V, N′, and V′ were observed. As shown in Figure 1A, the branches of wild-type plants (N′) are spread flat or oblique, while the branches of mutant plants (V′) were fascicled densely and spherical. The V′ was a dwarf, had multiple branches, and had shorter leaves than N′. Interestingly, the wild-type and mutant plant grafted seedlings (N and V) also show the same differences as N′ and V′ (Figure 1B).

**Figure 2:**
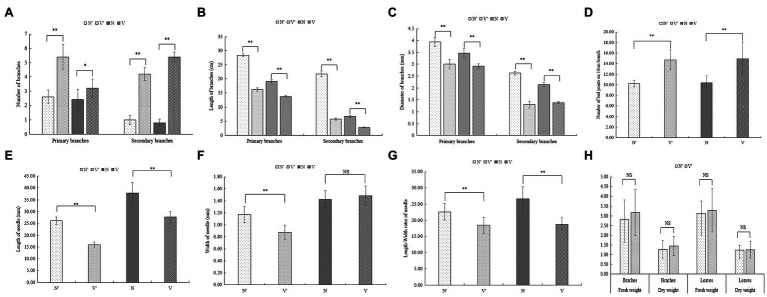
Comparison of growth traits between wild-type and mutant plants. **(A)** The number of branches. **(B)** The length of branches. **(C)** The width of branches. **(D)** The number of bud point on 10 cm branch. **(E)** The length of needle. **(F)** The width of needle. **(G)** The length-width ratio of needle. **(H)** The fresh and dry weight. The error bars represent standard error. ‘*’ on error bars indicate significant differences at *p* < 0.05, ‘**’ on error bars indicate significant differences at *p* < 0.01.

To further investigate the differences between wild-type and mutant plants, the growth traits of N′, V′, N, and V were evaluated. There were significant differences in the number of branches between wild-type and mutant plants (Figure 1C). The average number of primary and secondary branches of N′ and N was 2.6, 2.4, 1, 0.8, respectively. However, the average number of primary and secondary branches of V′ and V was 5.4, 3, 4.2, and 5.4, respectively (Figure 2A; Supplementary Table 1). Morphological observation and measurement results showed that there were significant differences between N′ and V′, N and V in branch length, width, and bud point number (Figure 1D). As shown in Figures 2B,C, the primary and secondary branch length and width of N and N′ were significantly higher than that of V and V′. The number of bud points on a 10 cm branch in V and V′ was significantly higher than that in N and N′ (Figure 2D; Supplementary Tables 1 and 2).

Moreover, we found that all needle characteristics were significantly different between V and V′ and N and N′ (Figures 1E, 2E, F, G; Supplementary Table 3). On measuring the variations in biomass, it was found that the fresh and dry weight of needles and stems in V and V′ were slightly higher than that of N and N′ (Figure 2H; Supplementary Table 4). All our physiological measurements confirm our initial selection of a mutant with smaller, shorter leaves and multiple branches. Furthermore, the difference between N′ and V′ is consistent with the difference between N and V, indicating that these variations were not caused by environmental factors.

### Changes in the Cytological Structure of Mutant Plants

The cytological structure of the buds, needles, and stems in N′ and V′ were observed as changes in the growth traits of mutant plants might be related to the development of plant tissue. As shown in Supplementary Figure 1, the differences in tracheid length, tracheid, and tracheid length-width ratio between N′ and V′, N and V were significant. The tracheid length and tracheid length-width ratio of V′ and V were significantly higher than that of N′ and N; however, the V′ and V tracheid width was significantly lower than that of N′ and N.

**Figure 3:**
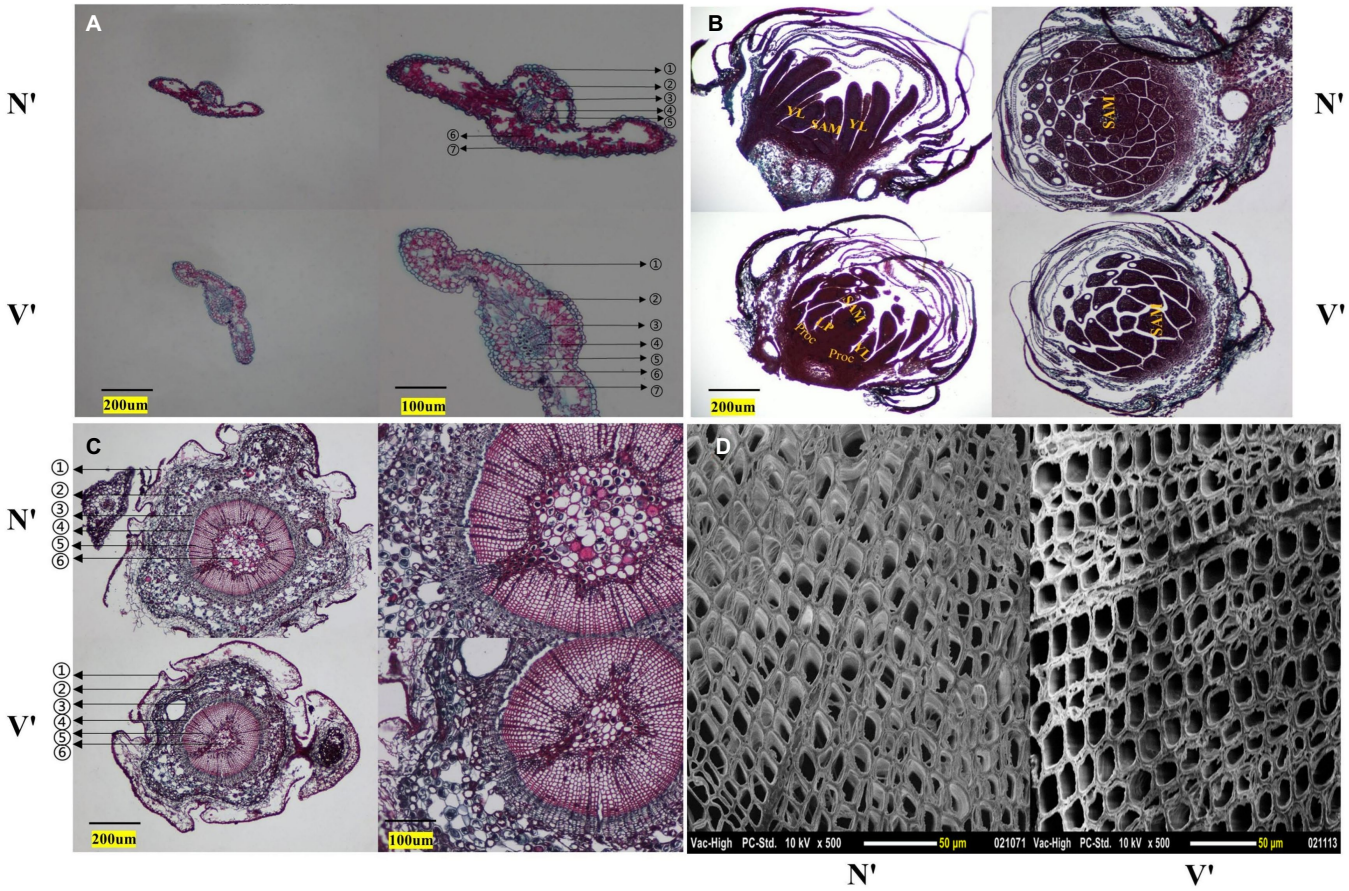
Cytological structure of wild-type and mutant plants. **(A)** Cross section structure of needles. ① stratum corneum; ② epithelial layer; ③ endodermis; ④ vascular bundle; ⑤ transfusion tissue; ⑥ mesophyll; ⑦ lower epidermis. **(B)** Longitudinal and cross section structures of buds. SAM, shoot apical meristem; YL, young leaf; LP, leaf primordium; Proc, procambium. **(C)** Cross section structure of annual stem. ① periderm; ② cortex; ③ secondary stem; ④ cambium; ⑤ secondary xylem; ⑥ pith. **(D)** Scanning electron microscope images of stem.

The microstructure of buds, needles, and stems in N′ and V′ was also observed. In the needles, the anatomical structure of mutant plant was different with wild-type plant. The endodermis, transfusion tissue, and lower epidermis of mutant plant showed obvious changes. The study found that the lower epidermis and transfusion tissue cells of mutant plant were larger than wild-type plant, but the endodermis cells of mutant plant were smaller than wild-type plant (Figure 3A). As shown in Figure 3B, in the microstructure of buds, the procambium, leaf primordium, and young leaves can be seen in the mutant plant. However, the wild-type plant only shows young leaves and almost no leaf primordium. As a rule, during the development of young leaves, the procambium first develops to form leaf primordia, and then, leaf primordia develops to form young leaves. This indicated that the buds of mutant plant were still in the stage of procambium development into leaf primordia, while the buds of wild-type plant had passed this stage and developed into young leaves. These results showed that the bud development of mutant plant is slower than that of wild-type plant. Furthermore, in the microstructure of stems, it was found that the proportion of xylem of mutant plant was smaller than wild-type plant, and the inner diameter and wall thickness of pith cells of mutant plant were also smaller than wild-type plant (Figure 3C, Supplementary Figures 2A,B). In addition, as shown in Figure 3D, the inner diameter of mutant plant tracheid cells was larger than wild-type plant, but the wall thickness of tracheid cells of mutant plant was smaller than wild-type plant (Supplementary Figures 2C,D).

### Changes in the Photosynthetic and Hormone Concentration of Mutant Plants

The dynamic photosynthetic rate (Pn), stomatal conductance (Gs), Transpiration rate (Tr), WUE, and intercellular CO2 concentration (Ci) were measured to examine the effects of bud mutation on photosynthesis. The results showed that Pn, Gs, Tr, and WUE of V were lower than that of N, while Ci of V was higher than that of N. There were significant differences in Pn, Ci, and WUE. However, no significant differences in Gs and Tr between N and V were observed (Supplementary Figures 3A–E).

To further investigate the difference between wild-type and mutant plants, the concentrations of four endogenous hormones were measured, including Indole-3-acetic acid (IAA), Gibberellic acid (GA), ABA and ethylene. As shown in Supplementary Figure 3F, the concentrations of IAA varied significantly in different months, different materials, and different tissues. In July (vigorous growth period), the IAA concentrations in the needles and shoots of V and V′ were lower than that of N and N′. In September (late growth period), the IAA concentrations in the needles of V and V′ were higher than that of N and N′, while the IAA concentrations in the shoots of V and V′ were lower than that of N and N′. Additionally, the data showed that the changing trend in GA and ABA concentrations was similar in July and September. The GA and ABA concentrations in V and V′ needles were higher than that of N and N′ needles, while the concentrations of GA and ABA in V and V′ shoots were lower than that of N and N′ shoots. In July and September, the ethylene concentrations in needles and shoots of V and V′ were significantly lower than that of N and N′ (Supplementary Figures 3G–I).

### RNA Sequencing and DEGs Analysis

To analyze the changes in gene expression patterns of bud mutations, RNA-seq was performed using the buds, needles and stems from N′ and V′. As a result, a total of 116.59 Gb clean data was obtained from 18 samples, and each sample amounted to 6.17 Gb. The average GC content was 46.56%, and Q30 percentages were all over 92.87% (Supplementary Table 5). The Trinity software was used for transcriptome assembly, and a total of 453,001 Contig were obtained, Contig N50 was 1,601 bp, the average length of a Contig was 882.7 bp, and the GC content was 41.48% (Supplementary Table 6). Particularly, 81.40% of the clean reads derived from the sequencing samples were mapped to the assembled sequences (Supplementary Table 7). BUSCO software was used to evaluate the assembly quality. A total of 1,375 genes were tested, among which BUSCO gene coverage reached 92.60% (1273), indicating the high level of assembly quality (Supplementary Table 8). A total of 78,740 unigenes were obtained after assembly, and then, the unigenes were aligned with sequences from common databases using BLAST for further functional annotations, including COG, GO, KEGG, KOG, Pfam, SwissProt and NR databases. A total of 33,215 unigenes were successfully annotated, accounting for 42.18% of the total number of unigenes. Among them, the number of unigenes (31,601) aligned to Nr was the largest, while the number of unigenes (9,504) aligned to COG was the lowest (Table 1).

**Table 1:** Unigene annotations statistics of *L. olgensis*.

Public database	Number	300 ≤ Length	Length ≥ 1,000
COG	9,504	2,173	5,919
GO	17,333	5,151	8,129
KEGG	10,705	2,980	5,874
KOG	18,402	5,659	8,824
Pfam	21,960	6,111	12,430
Swissport	20,219	6,056	10,900
eggNOG	28,229	8,415	14,120
Nr	31,601	10,148	14,926
All	33,215	10,877	10,877

**Figure 4:**
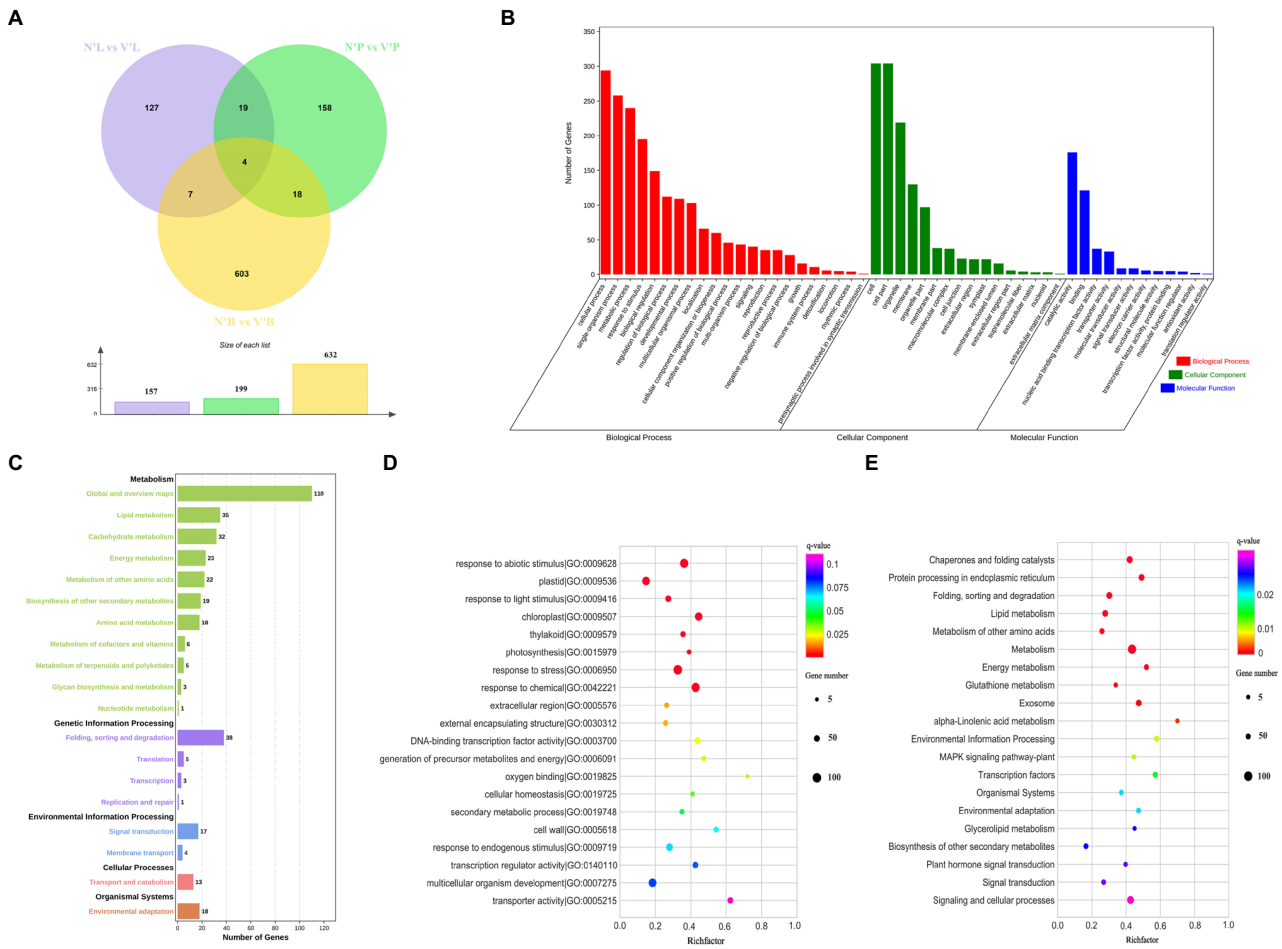
Functional annotation of differentially expressed genes (DEGs) in wild-type and mutant plants from *L. olgensis* parent trees. **(A)** Venn diagram of DEGs among different samples. **(B)** GO classification of DEGs. **(C)** Statistic analysis of DEGs in KEGG pathways. **(D)** The top 20 GO enrichment pathways of DEGs. **(E)** The top 20 KEGG enrichment pathways of DEGs.

To detect and evaluate the relative gene expression level among each sample, DEseq2 was used to identify DEGs with specified thresholds (Love et al., 2014). These samples contained buds, needles, and stems from wild-type and mutant plants (hereafter referred to as N’B, N’L, N’P, V’B, V’L, and V’P). All samples were divided into three comparison groups (N’B vs. V’B, N’L vs. V’L, and N’P vs. V’P), and each group has DEGs. The results showed that a total of 632 DEGs (423 upregulated and 209 downregulated), 157 DEGs (110 upregulated and 47 downregulated), and 199 DEGs (145 upregulated and 54 downregulated) were identified between N’B vs. V’B, N’L vs. V’L, and N’P vs. V’P, respectively (Figure 4A). In the three comparison groups, DEGs were higher in buds than in stems and leaves, which might be related to the mutants. In addition, a total of 11 genes were obtained in the overlap of DEGs between N’B vs. V’B and N’L vs. V’L samples, 22 genes were obtained in the overlap of DEGs between N’B vs. V’B and N’P vs. V’P samples, and 23 genes were obtained in the overlap of DEGs between N’L vs. V’L and N’P vs. V’P samples (Figure 4A).

### Gene Ontology and KEGG Pathway Analysis of DEGs

To understand the biological function of DEGs, functional classification of the identified DEGs was carried out using the GO annotation system. A total of 988 DEGs in N’B vs. V’B, N’L vs. V’L, and N’P vs. V’P were annotated in the biological process, cellular component, and molecular function. In the biological process, DEGs concentrated on the 22 terms, which mainly annotated in cellular process, single-organism process, and metabolic process. For the cellular component, DEGs concentrated on the 16 terms, which mainly annotated in cell, cell part, and organelle. For molecular function, DEGs mainly annotated the catalytic activities, binding, and nucleic acid binding transcription factor activity (Figure 4B and Supplementary Table 9). GO enrichment analysis was performed on the DEGs to obtain detailed functional information. The results showed that most genes (129) were significantly (*p* < 0.05) enriched in the term of “response to abiotic stimulus” (GO:0009628), 122 genes were significantly enriched in “response to chemical” (GO:0042221), and 63 genes were enriched in “response to endogenous stimulus” (GO:0009719). These results indicated that DEGs are mainly related to catalytic activity, nucleic acid binding transcription factor activity, and response to a stimulus (Figure 4D and Supplementary Table 10).

Furthermore, KEGG pathway analyses of DEGs in the three comparison groups were performed and five pathways were annotated, including metabolism, genetic information process, environmental information processing, cellular process, and organismal systems. In these pathways, “global and overview maps,” “lipid metabolism,” “carbohydrate metabolism,” and “energy metabolism” were considered as the most common pathways in metabolism. For genetic information processing, 43 DEGs were annotated in “folding, sorting, and degradation” and “translation” pathways. Moreover, most of the DEGs also were annotated in “signal transduction,” “membrane transport,” and “environment adaptation” pathways (Figure 4C and Supplementary Table 9). KEGG enrichment was performed to understand the major pathway of DEGs. The analysis indicated that “metabolism,” “signaling and cellular processes,” and “plant hormone signal transduction” were significantly enriched (value of *q* < 0.05) by DEGs. Moreover, “transcription factors,” “signal transduction,” and “biosynthesis of other secondary metabolites” were also enriched (Figure 4E and Supplementary Table 11). These results suggested that there are many changes in genes involved in plant hormone signal transduction, metabolic pathways, and signaling and cellular processes, which may play an important role in bud mutation.

### DEGs Involved in Cell Division, Cell Differentiation, and SAM Activity

**Figure 5:**
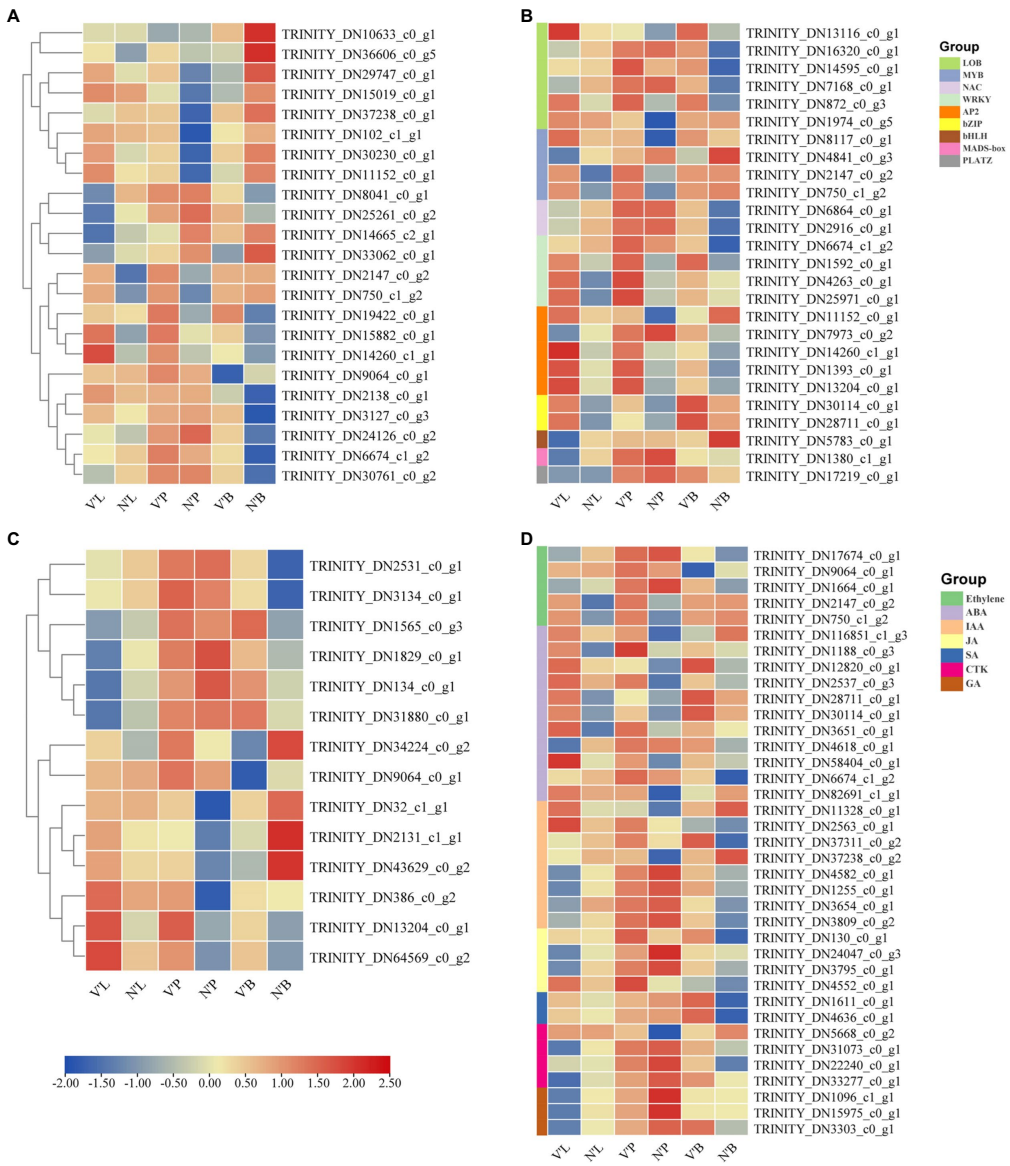
Heat map of relative expression levels of predicted DEGs involved in cell division and differentiation, transcription factors, sugar metabolism, and plant hormones. **(A)** DEGs involved in cell division and differentiation. **(B)** DEGs involved in transcription factors. **(C)** DEGs involved in sugar metabolism. **(D)** DEGs involved in plant hormones. Color scale represents the gene expression level.

Cell division, expansion, and differentiation affect the fundamental processes of plant organ growth and development and affect the plant phenotypes (Sugiyama, 2005; Yang et al., 2015). In addition, the shoot apical meristem (SAM) is essential for the development of plants and is responsible for the development of leaves, stems, and flowers (Han et al., 2019). Therefore, we analyzed 23 DEGs related to cell division, differentiation, and SAM activity to further study the differences between wild-type and mutant plants (Figure 5A). Among the 23 DEGs, 9 DEGs were annotated as cell division, 10 DEGs were involved in cell differentiation and cycle, and 4 DEGs were related to SAM activity. The results found that the expression levels of five genes (*TRINITY_DN2138_c0_g1*, *TRINITY_DN3127_c0_g3*, *TRINITY_DN24126_c0_g2*, *TRINITY_DN6674_c1_g2,* and *TRINITY_DN30761 _c0_g2*) in V’B were higher than that in N’B. However, the expression levels of four genes (*TRINITY_DN10633_c0_g1*, *TRINITY_DN36606_c0_g5*, *TRINITY_DN29747_c0_g1,* and *TRINITY_DN15019_c0_g1*) in V’B were lower than that in N’B. Compared with N’L and N’P, all the five genes (*TRINITY_DN2147_c0_g2*, *TRINITY_DN750_c1_g2*, *TRINITY_DN19422_c0_g1*, *TRINITY_DN15882_c0_g1*, and *TRINITY_DN14260_c1_g1*) were significantly expressed in V’L and V’P. In addition, the expression levels of the four DEGs (*TRINITY_DN8041_c0_g1*, *TRINITY_DN25261_c0_g2*, *TRINITY_DN14665_c2_g1,* and *TRINITY_DN33062_c0_g1*) in V’L were significantly lower than those in other tissues. These results showed that changes in these genes may be related to dwarf and multiple branches in mutant plants.

### DEGs Related to TFs

TFs play a vital role in plant development and gene expression regulation, forming complex gene regulatory networks (Muiño et al., 2016; Yang et al., 2018). In this study, a total of 27 DEGs belonged to genes encoding TFs and these genes belonged to nine TF families. As shown in Figure 5B, Six DEGs belonged to the LOB family; five DEGs in the AP2/ERF and MYB family; and one DEG in the HLH, MADS-box, and PLATZ family. Notably, all genes (*TRINITY_DN30114_c0_g1* and *TRINITY_DN28711_c0_g1*) of the bZIP family were significantly expressed in V’P, V’L, and V’B. In addition, three genes (*TRINITY_DN1592_c0_g1*, *TRINITY_DN4263_c0_g1*, and *TRINITY_DN25971_c0_g1*) from the WRKY family and three genes (*TRINITY_DN14260_c1_g1*, *TRINITY_DN1393_c0_g1,* and *TRINITY_DN13204_c0_g1*) from the AP2/ERF family were highly expressed in three tissues of mutant plants. Furthermore, the expression levels of many genes also changed in different tissues. For example, compared with N’L and N’P, *TRINITY_DN2147_c0_g2* and *TRINITY_DN750_c1_g2* of the MYB family were highly expressed in V’L and V’P, but the expression levels in N’B vs. V’B were almost unchanged. Particularly, compared with N’L, the expression levels of NAC, bHLH, and MADS-box family genes were decreased in V’L.

### DEGs Related to Sugar Metabolism

Sugar perception and signal transduction are important components that regulate plant growth and metabolism (Lei et al., 2011). Recent studies have reported the significant roles of sugar in plant growth and development (Wingler and Wingler, 2018; Luo et al., 2021). Thus, in this study, 14 DEGs associated with the sugar metabolism pathway and sugar and starch metabolism pathway was explored (Figure 5C). The expression level of the same gene differed among the different samples. Moreover, four genes (*TRINITY_DN1565_c0_g3*, *TRINITY_DN1829_c0_g1*, *TRINITY_DN134_c0_g1,* and *TRINITY_DN31880_c0_g1*) showed no significantly high expression in V’L but showed significantly high expression in V’P and V’B. Compared with N’P, *TRINITY_DN34224_c0_g2* and *TRINITY_DN9064_c0_g1* showed significantly high expression in V’P, whereas compared with N’B, these two genes showed significantly decreased expression in V’B. Additionally, three genes (*TRINITY_DN386_c0_g2*, *TRINITY_DN13204_c0_g1*, and *TRINITY_DN64569_c0_g2*) showed significantly increased expression in all tissues (leaf, stem, and bud) of the mutant plant (V′) compared with the wild-type plant (N') and had an upregulated expression profile.

### DEGs Involved in Plant Hormones

Since the mutant plant was a dwarf, had multiple branches, and had smaller leaves than wild-type plant, we hypothesized that key plant hormones might affect the tissue development of the mutant plant, which results in different phenotypic characteristics compared with the wild-type plant (Tian et al., 2021b). Therefore, 37 DEGs related to ethylene signaling pathway, ABA metabolic process, ABA signaling pathway, IAA signaling pathway and GA biosynthetic process were identified to analyze the expression changes in the three comparison groups. These identified hormones belong to seven families, ABA with 11 DEGs was the largest of the families, followed by IAA with 8 DEGs, ethylene with 5 DEGs, JA and CTK with 4 DEGs, GA with 3 DEGs, and SA with only 2 DEGs (Figure 5D). The study found that two genes (*TRINITY_DN2147_c0_g2* and *TRINITY_DN750_c1_g2*) participating in the IAA signaling pathway showed increased expression in V’L and V’P, but showed decreased expression in V’B. Compared with N’B, the expression levels of genes (*TRINITY_DN1611_c0_g1* and *TRINITY_DN4636_c0_g1*) involved in the salicylic acid metabolic process were significantly increased in V’B. In addition, compared with N’L, the expression levels of three genes (*TRINITY_DN1096_c1_g1*, *TRINITY_DN15975_c0_g1,* and *TRINITY_DN3303_c0_g1*) related to the GA biosynthetic process were decreased in V’L.

**Figure 6:**
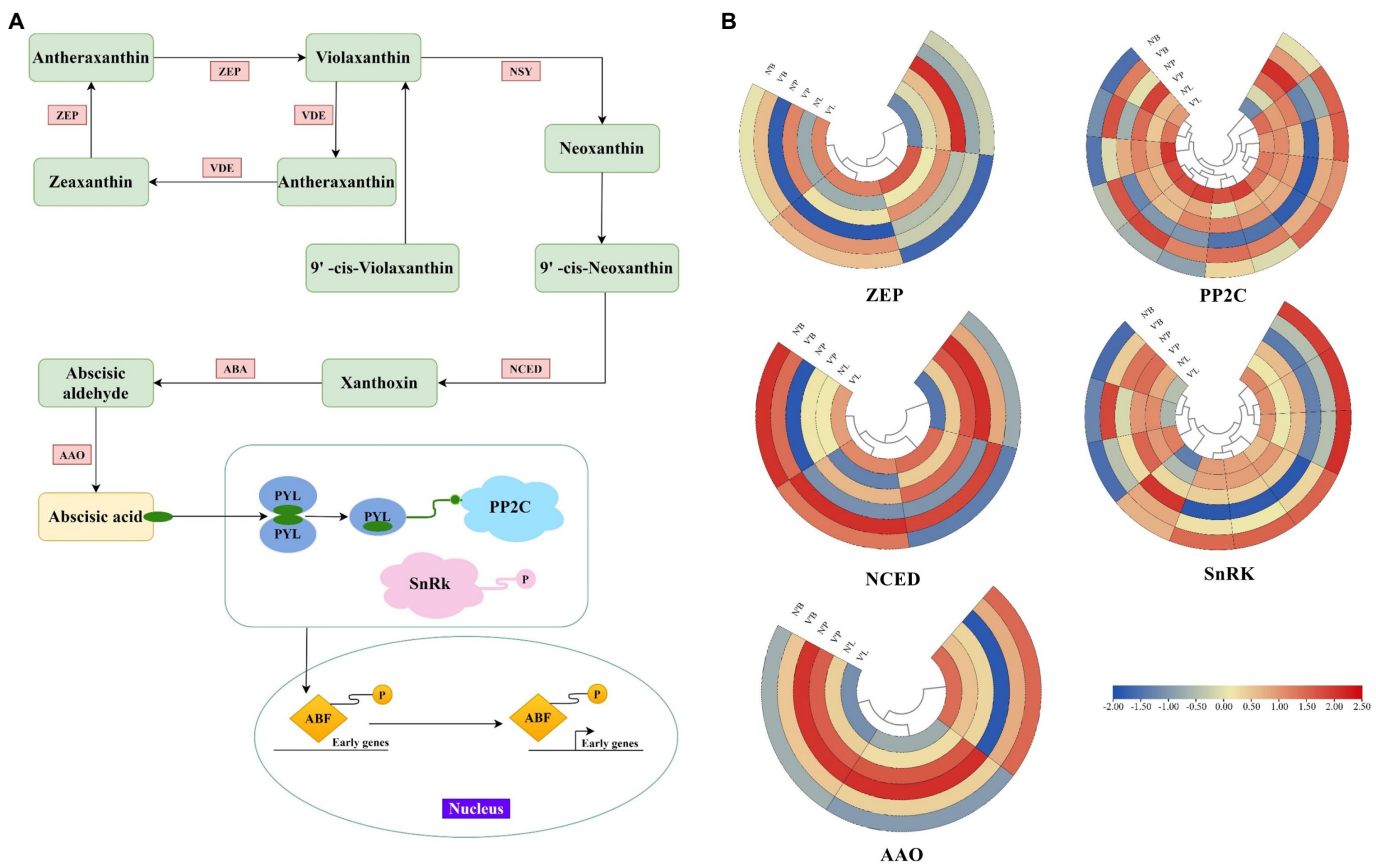
Expression profiles of DEGs associated with abscisic acid (ABA). **(A)** Biosynthetic and signal transduction pathways of ABA. Pathway was constructed based on the KEGG pathway and literary references. ZEP, zeaxanthin epoxidase; NSY, neoxanthin synthase; VDE, violaxanthin de-epoxidase; ABA, xanthoxin dehydrogenase; NCED, 9-cis-epoxycarotenoid dioxygenase; AAO, abscisic-aldehyde oxidase; PYL, pyrabactin-resistance1-like; PP2C, protein phosphatase type 2C; SnRK, serine/threonine-protein kinase; ABF, ABA responsive element binding factor. **(B)** Heat map of relative expression levels of DEGs involved in ABA biosynthetic and signal transduction pathways, including ZEP, NCED, AAO, PP2C, and SnRK family. Color scale represents the gene expression level.

ABA is a key phytohormone that regulates various aspects of plant development (Arend et al., 2009). In this study, except for *TRINITY_DN4618_c0_g1* and *TRINITY_DN116851_c1_g3*, all genes associated with the ABA metabolic process were highly expressed in three mutant plant tissues, and ABA was also found to be the largest hormone species with 11 DEGs. Therefore, to further investigate plant hormone expressions in mutant plants, we examined the genes involved in ABA synthesis and the ABA signaling pathway (Figure 6A). A total of 34 key DEGs were detected in the ABA synthesis pathway, including the zeaxanthin epoxidase (ZEP), 9-cis-epoxycarotenoid dioxygenase (NCED), abscisic-aldehyde oxidase (AAO), protein phosphatase type 2C (PP2C), and serine/threonine-protein kinase (SnRK) family genes (Figure 6B). Among them, the largest family was PP2C with 13 DEGs, followed by SnRK with 10 DEGs, ZEP and NCED with 4 DEGs, and AAO with 3 DEGs (Supplementary Table 12). In the ZEP family, *TRINITY_DN2802_c0_g2* and *TRINITY_DN1088_c0_g2* had increased expression levels in V’L, while *TRINITY_DN281_c0_g3* had decreased expression in V’L. Two genes (*TRINITY_DN9920_c1_g1* and *TRINIT_DN11756_c0_g2*) related to the NCED family were highly expressed in all tissues of V'. The expression levels of *TRINITY_DN18606_c0_g1* and *TRINITY_DN3795_c0_g3* associated with the AAO family were significantly high in N’P and V’P. In addition, among ABA signaling genes, 9 genes were upregulated in the three comparison groups, including 8 genes (*TRINITY_DN6094_c0_g1*, *TRINITY_DN849_c1_g1*, *TRINITY_DN13589_c0_g1*, *TRINITY_DN423 _c6_g1*, *TRINITY_DN11756_c0_g1*, *TRINITY_DN14738_c0_g2*, *TRINITY_DN12820_c0_g2,* and *TRINITY_DN54662_c0_g1*) in the PP2C family and 1 gene (*TRINITY_DN5719_c0_g1*) in the SnRK family.

### Quantitative Real-Time Polymerase Chain Reaction Validation

To evaluate the reliability of the sequencing results, 16 DEGs (5 DEGs from N’L vs. V’L, 5 DEGs from N’B vs. V’B, and 6 DEGs from N’P vs. V’P) were randomly selected and analyzed using qRT-PCR. As shown in Supplementary Figure 4, the qRT-PCR expression trend of the 16 DEGs was consistent with the RNA-seq results. The qRT-PCR results proved that the expression profile results of RNA-seq were true and reliable. Primers for qRT-PCR are shown in the supplementary files (Supplementary Table 13).

## Discussion

With the development of science and technology, plant mutation studies have been receiving attention, even though the mechanisms of mutation in some species remain unclear (Uematsu et al., 2014; Zhang et al., 2020b). *L. olgensis* is a tall deciduous tree species, it is a fast-growing tree with strong adaptability, high ornamental value, and excellent wood properties (Song et al., 2021). However, due to the long growth cycle, complex genetic background, and low natural conifer mutation frequency, few studies report about the mutant of *L. olgensis*. In the present study, we found that the *L. olgensis* mutant plants had the following traits: dwarf, short leaves, and multiple branches, which is significantly different from the wild-type plant. Unfortunately, the molecular regulatory mechanisms of *L. olgensis* mutant plants remain unknown. Therefore, the physiological, and biochemical index of wild-type and mutant plants of *L. olgensis* were statistically analyzed, and the cytological structures were observed. Additionally, the transcriptome sequencing of different tissues in the wild-type and mutant plants was performed to find candidate genes contributing to bud mutation, which laid the foundation for the subsequent study of bud mutation mechanism in *L. olgensis*.

### Changes in Morphological and Cytological Structures of Mutant Plants

Morphological identification is the most common and direct method to determine bud mutation. In this study, the morphological differences between the mutant and wild-type plants of *L. olgensis* were distinguishable. The study results showed that the mutant plant was characterized by multiple branches, short branches, and short leaves, while the wild-type plant was characterized by few branches, long branches, and long leaves. Similar results were also reported in few other plants, such as physic nut (Chen et al., 2019) and birch (Han et al., 2019). In addition, the observational and statistical analyses of mutant grafted seedlings showed that the variant traits could be retained in grafted plants. Similar results are consistent with the previously reported results for sweet orange (Marcotrigiano, 1997; Liu et al., 2009). These results suggest that the new phenotype of mutants may be maintained and inherited through asexual reproduction of offspring. These excellent mutant plants can provide new and useful genetic information for further genetic improvement of *L. olgensis*.

Tracheid, which is smaller and thicker than the conduit in angiosperms, is the only water-carrying tissue in the xylem of gymnosperms (Nardini et al., 2007). Various studies have reported that different tracheid widths and lengths can affect the water transport resistance of the xylem (Rolland et al., 2015). A small width and short length of tracheid will increase water transport resistance and decrease water transport efficiency (Trifilò et al., 2010; Tumajer and Treml, 2019). In this study, compared with the wild-type plant, the mutant plant had a longer tracheid length, smaller tracheid wall thickness, and larger tracheid width. These results may indicate that mutant plants have lower water transport resistance and higher water transport efficiency compared with the wild-type plants, which may be the reason for the presence of multiple branches in mutant plants.

### Changes in Physiological Characteristics of Mutant Plants

Photosynthesis is a fundamental process of plant growth and development, and the physiological indexes related to photosynthesis effectively reflect the photosynthetic capacity of plants (Guo et al., 2020). In this study, five indexes (Pn, Gs, Tr, WUE, and Ci) were measured to investigate the effect of bud mutation on photosynthesis. Studies have shown that changes in Gs and Ci mainly lead to the reduction of Pn, and when Ci and Gs decrease simultaneously, stomatal conductance will mainly restrict Pn. By contrast, if Ci increases as Gs decreases or remains constant, the decrease of Pn might be caused by non-stomatal factors (Song et al., 2014). The results found that Pn, Gs, and Tr of mutant plants were lower than wild-type plants, whereas the Ci of the mutant plant was higher than the wild-type plant. These results indicating that non-stomatal factors may be the main reason for the varied traits in mutant plants. Non-stomatal factors including the change in chloroplast structure, plant membrane system, and changes in various enzyme activities (Xie et al., 2014); therefore, we hypothesized that the changes in non-stomatal factors may account for the different phenotypes of mutant and wild-type plants.

Plant growth and development are highly related to the phytohormone content level and phytohormones ratios (Zhang et al., 2020a). In this study, plant hormone levels were significantly different between mutant and wild-type plants. During the vigorous growth period (July), the IAA concentrations in V and V′ needles were lower than that in N and N′, whereas, in the late growth period (September), the IAA concentrations in V and V′ needles were higher than that in N and N’. These results suggest that changes in endogenous hormones might be caused by seasonal changes in mutant plants that further affect plant growth and development. The result was similar to previous studies on *Betula pubescens* (Rinne et al., 1994), and a study of *Abelmoschus Esculentus* (Aminu et al., 2019) showed that the concentration of endogenous hormones in plant tissues is related to seasonal change. Moreover, in July and September, GA and ABA concentrations in V and V′ needles were higher than that in N and N’. These results indicate that there may be different growth patterns between the mutant and wild-type plants. According to the changes in hormone concentration, we speculated that the growth rate of the mutant plant was slower than the wild-type plant in the vigorous growth period but faster than that of the wild-type plant during the late growth period. Therefore, we hypothesized that the differences in hormone concentrations may be the reason for the differences between mutant and wild-type plants, and the high hormone concentration in the mutant plant may affect the presence of multiple branches.

### Changes in Genes Associated With Cell Differentiation in the Mutant Plants

Plant morphological development is closely related to various genes that control cell division, expansion, and differentiation (Lopez-Hernandez et al., 2020). In addition, the branching structure of plants is derived from SAM during embryonic development (Bi et al., 2019). Previous studies reported that the transcription mechanism of the inflorescence branching mutants in *Jatropha curcas* is closely related to genes involved in cell division, cell differentiation, and flower development (Chen et al., 2019). Another study of woodland strawberry mutant reported that the differential expression of genes related to cell division, cell differentiation, and SAM activity was the main factor affecting fruit size (Wang et al., 2017). This study provides further evidence supporting other previously reported findings.

In this study, 23 DEGs were associated with cell division, cell differentiation, and SAM activity, but many genes were expressed at low levels in mutant plants. For example, two genes (*TRINITY_DN33062_c0_g1* and *TRINITY_DN8041_c0_g1*) expression levels in V’L were significantly lower than that in other tissues. Annotation analysis revealed that the two genes encoding the homeobox-leucine zipper protein and GPI-anchored protein, and we hypothesized that they may affect the synthesis of related proteins and, thus, affect cell differentiation in mutant plants. In addition, the expression levels of the 5 genes (*TRINITY_DN2138_c0_g1*, *TRINITY_DN3127_c0_g3*, *TRINITY_DN24126_c0_g2*, *TRINITY_DN6674_c1_g2*, and *TRINITY_DN30761_c0_g2*) in V’B were higher than those in N’B, suggesting that these genes may be related to bud differentiation and play a key role in mutant plant. These results suggest that the differential expression of genes related to cell division, cell differentiation and SAM structure may be the reason for the difference between mutant and wild-type plants, and thus, new phenotypes of mutant plants were produced.

### Changes in TFs in the Mutant Plants

TFs regulate gene expression by binding to cis-acting elements in the promoter region and play an important role in plant structure formation and development (Jiao et al., 2018; Li et al., 2021). Currently, TFs have been reported that control the development of bud and lateral branches. The LOB family has been reported to participate in the morphogenesis of lateral organs and the establishment of apical meristem boundaries in *Arabidopsis* (Chalfun-Junior et al., 2005; Lee et al., 2019). Additionally, it has been reported that the MYB family directly regulates the development of lateral meristem in *Arabidopsis* and tomato (Müller et al., 2006; Han et al., 2018). These reports indicate that these TFs are closely related to the branch formation process. In this study, the expression levels changed in some TFs, a total of 27 DEGs encoded nine TF families, including the LOB, AP2/ERF, and MYB families. Among them, the LOB family was the largest TF family with 6 DEGs, which may play an important role in the multiple branch formation of mutant plants. This is consistent with previous studies in *Arabidopsis*. Moreover, our study identified more TFs than previously reported, suggesting the role of a more complex transcriptional regulation network for mutant plant development (Yang et al., 2018). Particularly, the expression levels of genes from the same TF families varied in different samples, which was consistent with a previously reported study on flowering traits of *Liriodendron chinense* mutant (Sheng et al., 2021). These results suggest that compared with wild-type plants, some DEGs encoding transcription factor may play a role in mutant development and affect the generation of specific traits, leading to differential traits between wild-type and mutant plants.

### Changes in Genes Associated With Sugar Metabolism in the Mutant Plants

In plants, sugar plays an essential role in growth and development. Soluble sugar accumulation is a common phenomenon during plant development (Yang et al., 2019). Sugar acts as a signaling molecule that transmits the cell’s metabolic status to regulate plant growth and development (Debast et al., 2011; Cai et al., 2021). Beveridge et al. proved that the initial signal for lateral bud growth was not auxin, but sucrose (Beveridge et al., 2000). Mason et al. pointed out that sucrose treatment could significantly promote lateral bud growth and downregulate lateral bud inhibitory gene expression (Mason et al., 2014). In this study, a total of 14 DEGs associated with sugar and starch metabolism pathways were explored. Among them, two genes (*TRINIT_DN64569_c0_g2* and *TRINITY_DN386_c0_g2*) encoding sucrose phosphate synthase were upregulated in all mutant plant tissues, indicating a possible role of sucrose biosynthesis in mutant plant-specific traits. The result was consistent with previous studies on *Arabidopsis thaliana* (Kebrom and Mullet, 2015), and a study of *Brassica pekinensis* (Xia et al., 2015) showed that sucrose plays a key regulatory role in branching development and flowering. Additionally, the expression level of a sugar signal transduction gene (*TRINITY_DN13204_c0_g1*) was significantly increased in mutant plant tissues, which is consistent with previous reports suggesting that sugar can be used as a signaling molecule and sugar levels can be trigger factors for lateral bud development (Smeekens et al., 2010; Granot et al., 2013). Furthermore, two genes involved in the starch metabolism pathway were also found to be involved in mutant development, suggesting that the genes encode sugar and starch metabolism are associated with mutant plant development. These results indicate that the expression levels of genes related to sugar metabolism are different between wild-type and mutant plants. Many genes related to glucose metabolism are highly expressed in mutant plants, which may be the factors influencing the differences between mutant and wild-type plants.

### Changes in Plant Hormone Genes in the Mutant Plants

Plant hormones are essential for many fundamental and developmental processes of plants, such as cell division, bud development, shoot branching, and senescence (Heyl et al., 2007). Genes related to hormonal metabolism and signal transduction also play important roles in regulating plant and organ size (Guo et al., 2010). In this study, we identified 37 DEGs associated with plant hormones, which belong to seven hormone families. Among them, the expression levels of genes associated with cytokinin differed significantly in various tissues, indicating its role in cell division, cell development, and plant branching. This is consistent with reports concluding that cytokinin directly promotes plant branching, tillering development, and lateral bud growth (Hutchison and Kieber, 2002; Foo et al., 2007). GA plays an important regulatory role in plant growth and development (Mao et al., 2017). GA is also known to control longitudinal shoot growth and shoot branching (Arend et al., 2009). In this study, 3 DEGs related to GA were found to have different expression levels in mutant and wild-type plants, suggesting that GA may play a role in mutant plants development.

ABA was the largest hormone species, with 11 DEGs, which are highly expressed in all tissues of the mutant plant. Combined with our physiological results on plant hormone concentration, ABA concentration was higher in mutant plant leaves than that of normal leaves, which was consistent with the RNA-seq results. Therefore, we hypothesized that ABA plays a more important role than other hormones in the differential changes between mutant and wild-type plants. ABA has been widely investigated as a major endogenous factor involved in seed germination inhibition, internode elongation, and bud dormancy (Da Silva et al., 2008; Singh et al., 2018). Our results indicate that the genes related to ABA are highly expressed in mutant plants compared with wild-type plants, which may inhibit the tissue development of mutant plants, resulting in dwarfed plants, short branches, and short leaves. In ABA biosynthesis, our results indicated that for ABA biosynthesis genes (ABA, AAO, and NCED) were upregulated in the mutant plant tissues, adding to our viewpoint. Similar patterns were also found in previous studies that stated ABA is closely related to plant tissue dormancy (Yin et al., 2009; Porcel et al., 2014). Furthermore, the expression levels of ABA signaling genes (PYL, PP2C, and SnRk) were also significantly changed in mutant plants compared with wild-type plants, again suggesting that ABA may inhibit mutant development. These results suggest that plant hormones may be the main regulatory factors of differential growth and development between mutant and wild-type plants, and ABA-related genes play a more important role in the variation of traits between mutant and wild-type plants.

### The Potential Value of Mutant Plants of *L. Olgensis* in Genetics and Breeding

**Figure 7:**
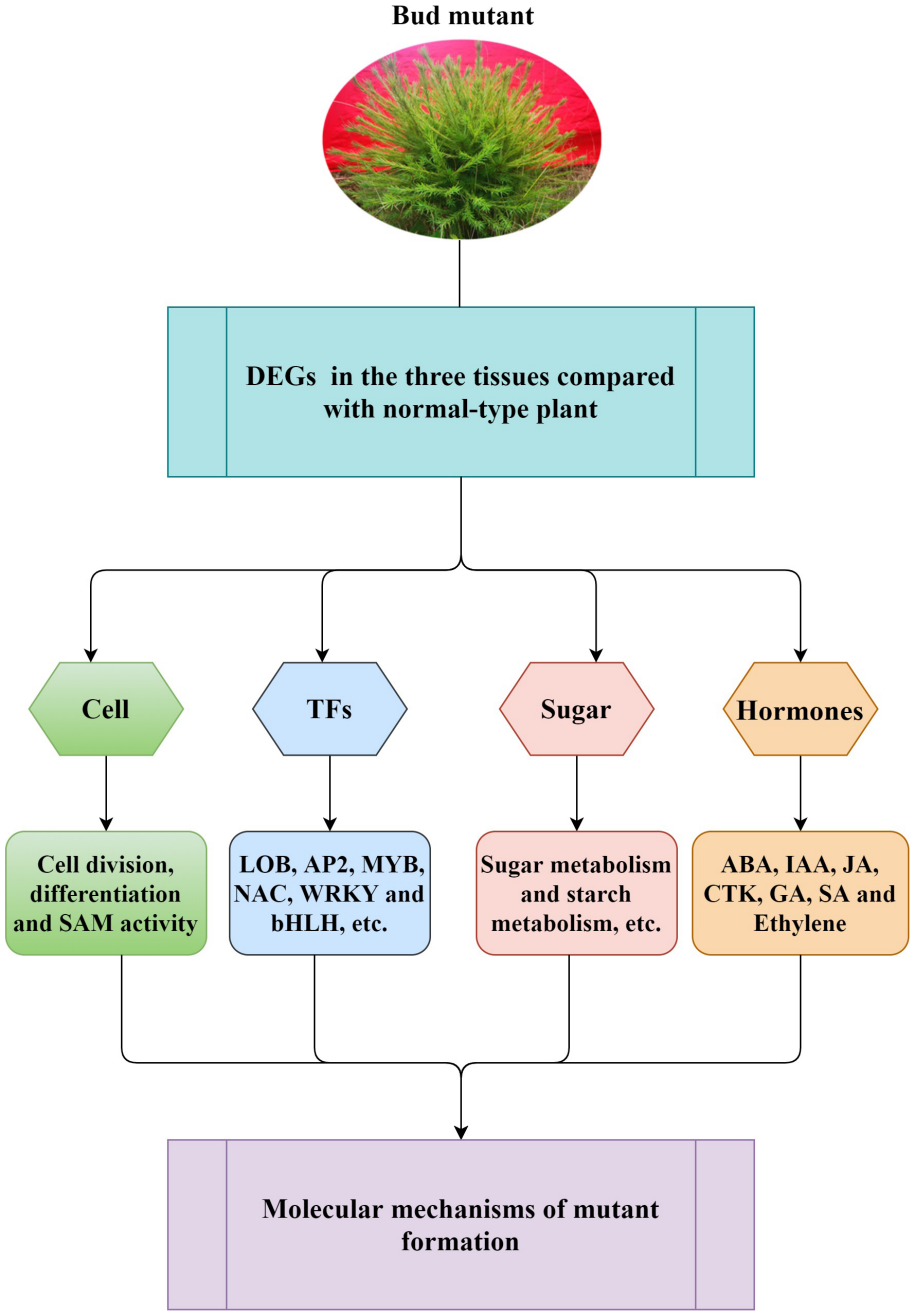
Schematic diagram of general molecular regulation mechanism for mutant formation. The model suggested that the differential expression of genes involved in cell division and differentiation, SAM activity, plant hormone biosynthesis, and sugar metabolism was closely related to the formation of *L. olgensis* mutants. Transcription factors have also been found to play a role in mutant formation.

A long breeding cycle and complex genetic background of *L. olgensis* are the main reasons for its slow genetic improvement. The rare natural bud mutation is convenient for breeding new varieties and the genetic improvement of *L. olgensis*. It also acts as a model plant for studying the molecular mechanism of conifer mutation. We have confirmed the special traits in mutant plants can be stably maintained by asexual reproduction methods such as grafting and cutting. Moreover, on investigating the molecular mechanisms associated with bud mutation (Figure 7), we found that the differential expression of genes involved in cell division and differentiation, SAM activity, plant hormone biosynthesis, and sugar metabolism were closely related to differential trait formation between mutant and wild-type plants. Understanding the molecular mechanism of multiple branch mutants lays the foundation for new variety breeding and genetic improvement of *L. olgensis*. For a long time, *L. olgensis* has been used only as an afforestation timber species. However, this study highlights that the mutants of *L. olgensis* are not only precious germplasm resources but also have potential agronomic value. These mutant plants have multiple branches, clustered branches, short leaves, spherical crowns, and high ornamental value, which can be planted as a new tree species for landscaping.

## Data Availability Statement

RNA-Seq data from wildtype and mutant plants samples were deposited in China National Center for Bioinformation (National Genomics Data Center) under the accession number CRA004862. The assembled unigenes were deposited in China National GeneBank (CNGBdb) under the accession number CNA0036280. The functional annotations and FPKM values of transcripts were deposited in Supplementary Tables 14 and 15.

## Author Contributions

KC was a major contributor in writing the manuscript. XuZ drafted the manuscript and substantially revised it. XL, YK, and XY analyzed the data and make figures. YC and GL participated in RNA extraction and performed qRT-PCR assay. XP and XiZ conceived of the study, participated in its design and data interpretation, and revised the manuscript critically. All authors have read and approved the final manuscript.

## Funding

This work was supported by the Scientific research start-up funds of Jilin Agricultural University (No. 2021002).

## Conflict of Interest

The authors declare that the research was conducted in the absence of any commercial or financial relationships that could be construed as a potential conflict of interest.

## Publisher’s Note

All claims expressed in this article are solely those of the authors and do not necessarily represent those of their affiliated organizations, or those of the publisher, the editors and the reviewers. Any product that may be evaluated in this article, or claim that may be made by its manufacturer, is not guaranteed or endorsed by the publisher.

## Footnotes

^1^ http://eggnog-mapper.embl.de/

^2^ https://wego.genomics.cn/

^3^ https://www.omicshare.com/tools
