# Supplementary material for: Insight Into the Multiple Branches Traits of a Mutant in Larix olgensis by Morphological, Cytological, and Transcriptional Analyses
Source: Front Plant Sci. 2021 Dec 21;12:787661. doi: 10.3389/fpls.2021.787661 (PMC8724527; doi:10.3389/fpls.2021.787661)
Supplement: Supplementary file 1 [file Data_Sheet_1.docx]

Supplementary Material

# Supplementary Figures and Tables

## Supplementary Figures


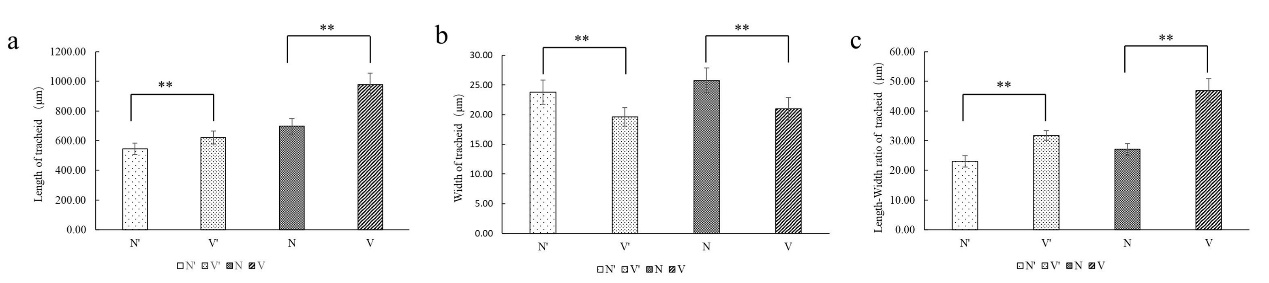


**Supplemental Figure 1.** Comparison of tracheid morphology between wild-type and mutant plants. (a) The length of tracheid. (b) The width of tracheid. (c) The length-width ratio of tracheid. The error bars represent standard error. ‘*’ on error bars indicate significant differences at *p* < 0.05, ‘**’ on error bars indicate significant differences at *p* < 0.01.


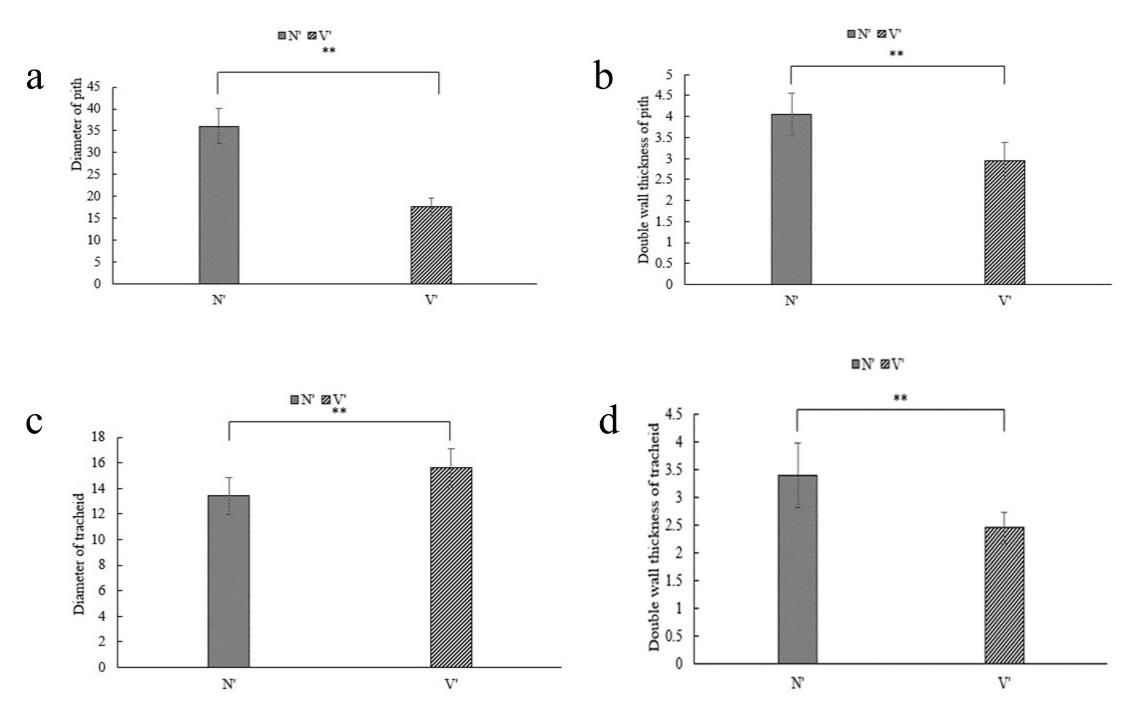


**Supplemental Figure 2.** Comparison of pith cell and tracheid morphology between wild-type (N') and mutant plants (V') from *L. olgensis* parent trees. (a) The inside diameter of pith cell. (b) The double wall thickness of pith cell. (c) The inside diameter of tracheid. (d) The double wall thickness of tracheid. The error bars represent standard error. ‘*’ on error bars indicate significant differences at *p* < 0.05, ‘**’ on error bars indicate significant differences at *p* < 0.01.


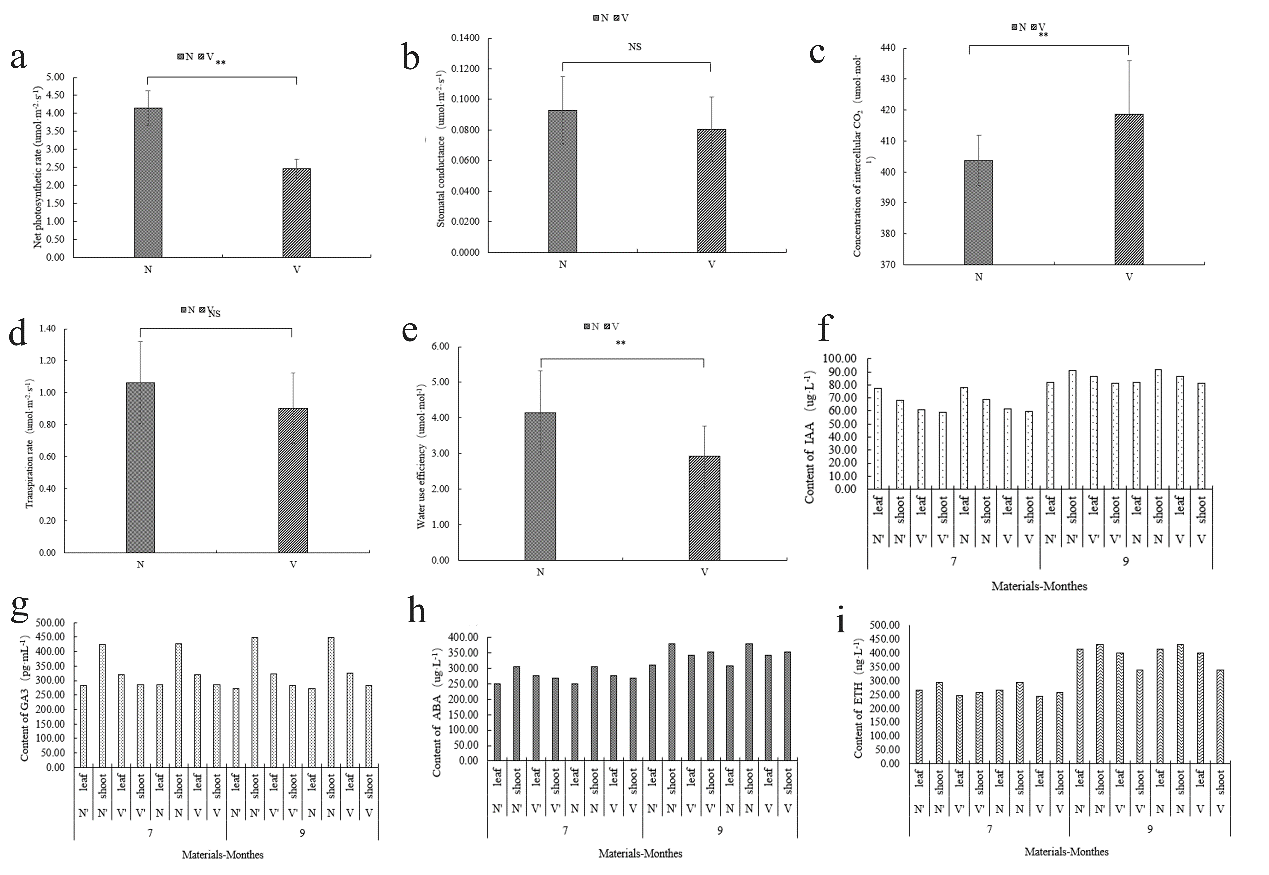


**Supplemental Figure 3.** Comparison of photosynthetic indicators and hormone content between wild-type and mutant plants. (a) Net photosynthetic rate. (b) Stomatal conductivity. (c) Intercellular carbon dioxide concentration. (d) Transpiration rate. (e) Water use efficiency. (f) IAA content. (g) GA3 content. (h) ABA content. (i) ETH content. The error bars represent standard error. ‘*’ on error bars indicate significant differences at *p* < 0.05, ‘**’ on error bars indicate significant differences at *p* < 0.01.


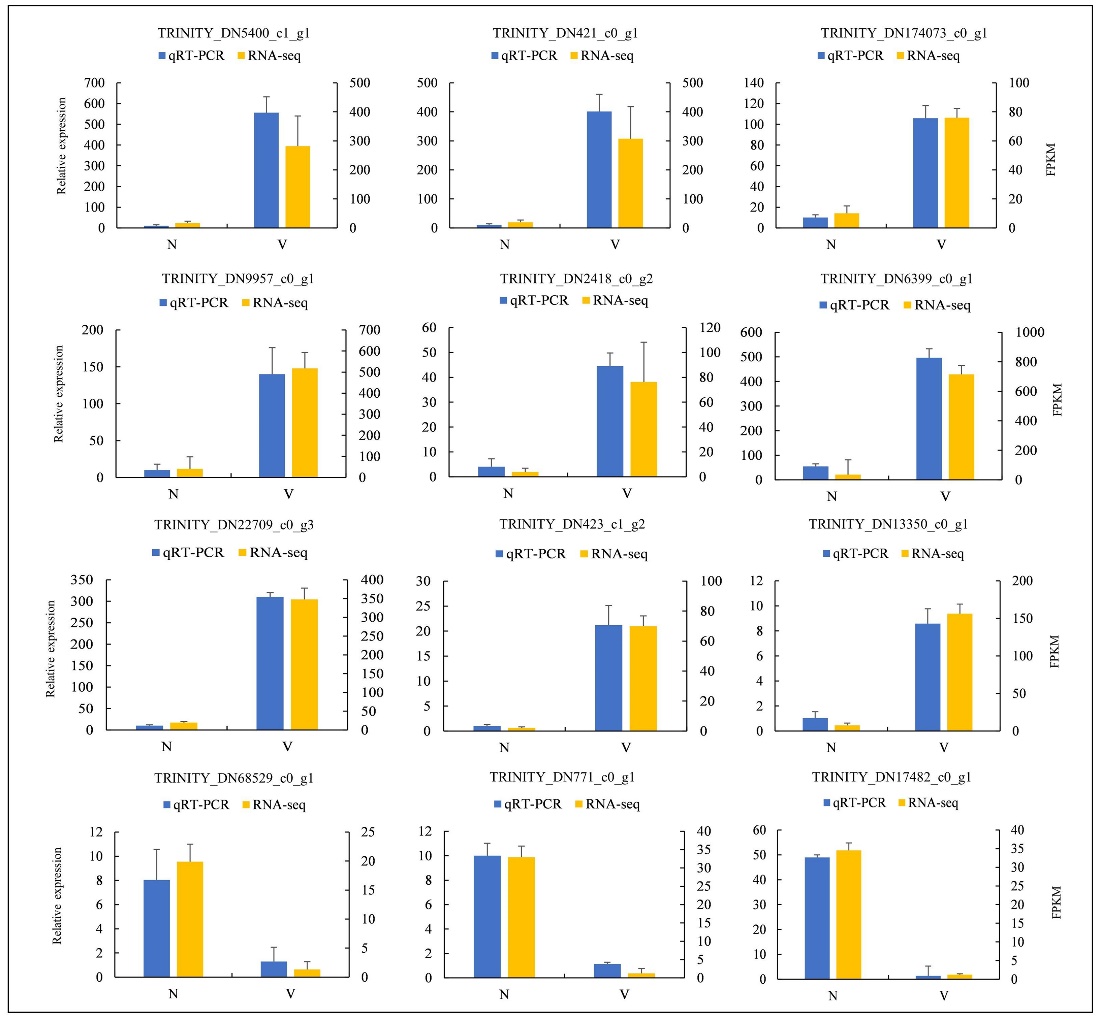


**Supplemental Figure 4**. qRT-PCR verification of expression level of 16 DEGs obtained by RNA sequencing. The X-axis represents the wild-type (N) and bud mutant (V) plants. The Y-axis on the left represents the expression data of qRT-PCR. The Y-axis on the right represents the relative expression levels of DEGs validated by RNA-seq.

## Supplementary Tables

**Table S1**: Comparison of branches growth traits between wild-type and mutant plants

**Table S2**: Comparison of bud point numbers between wild-type and mutant plants

**Table S3**: Comparison of needles growth traits between wild-type and mutant plants

**Table S4**: Comparison of fresh and dry weight between wild-type and mutant plants

**Table S5**: Sample sequencing data evaluation statistics.

**Table S6:** Unreferenced transcriptome assembly data statistics.

**Table S7**: Comparison of sequencing data and assembly results.

**Table S8**: BUSCO assessment results.

**Table S9**: Functional annotation of differentially expressed genes (DEGs).

**Table S10**: The top 20 GO enrichment pathways of differentially expressed genes.

**Table S11**: The top 20 KEGG enrichment pathways of differentially expressed genes.

**Table S12**: DEGs involved in ABA synthesis pathway, including ZEP, NCED, AAO, PP2C and SnRK family.

**Table S13**: Specific primers used for qRT-PCR analysis.

**Table S14**: FPKM values of all transcripts.

**Table S15**: The functional annotations of longest transcripts.
